# The ERI-6/7 Helicase Acts at the First Stage of an siRNA Amplification Pathway That Targets Recent Gene Duplications

**DOI:** 10.1371/journal.pgen.1002369

**Published:** 2011-11-10

**Authors:** Sylvia E. J. Fischer, Taiowa A. Montgomery, Chi Zhang, Noah Fahlgren, Peter C. Breen, Alexia Hwang, Christopher M. Sullivan, James C. Carrington, Gary Ruvkun

**Affiliations:** 1 Department of Molecular Biology, Massachusetts General Hospital, Department of Genetics, Harvard Medical School, Boston, Massachusetts, United States of America; 2 Center for Genome Research and Biocomputing, Oregon State University, Corvallis, Oregon, United States of America; Stanford University Medical Center, United States of America

## Abstract

Endogenous small interfering RNAs (siRNAs) are a class of naturally occuring regulatory RNAs found in fungi, plants, and animals. Some endogenous siRNAs are required to silence transposons or function in chromosome segregation; however, the specific roles of most endogenous siRNAs are unclear. The helicase gene *eri-6/7* was identified in the nematode *Caenorhabditis elegans* by the enhanced response to exogenous double-stranded RNAs (dsRNAs) of the null mutant. *eri-6/7* encodes a helicase homologous to small RNA factors Armitage in *Drosophila*, SDE3 in *Arabidopsis*, and Mov10 in humans. Here we show that *eri-6/7* mutations cause the loss of 26-nucleotide (nt) endogenous siRNAs derived from genes and pseudogenes in oocytes and embryos, as well as deficiencies in somatic 22-nucleotide secondary siRNAs corresponding to the same loci. About 80 genes are *eri-6/7* targets that generate the embryonic endogenous siRNAs that silence the corresponding mRNAs. These 80 genes share extensive nucleotide sequence homology and are poorly conserved, suggesting a role for these endogenous siRNAs in silencing of and thereby directing the fate of recently acquired, duplicated genes. Unlike most endogenous siRNAs in *C. elegans*, *eri-6/7*–dependent siRNAs require Dicer. We identify that the *eri-6/7*–dependent siRNAs have a passenger strand that is ∼19 nt and is inset by ∼3–4 nts from both ends of the 26 nt guide siRNA, suggesting non-canonical Dicer processing. Mutations in the Argonaute ERGO-1, which associates with *eri-6/7*–dependent 26 nt siRNAs, cause passenger strand stabilization, indicating that ERGO-1 is required to separate the siRNA duplex, presumably through endonucleolytic cleavage of the passenger strand. Thus, like several other siRNA–associated Argonautes with a conserved RNaseH motif, ERGO-1 appears to be required for siRNA maturation.

## Author Summary

Endogenous small interfering RNAs (siRNAs) are a class of small RNAs present in fungi, plants, and animals. Small RNAs, including microRNAs, are known to regulate the expression levels of genes, silence invading elements such as transposons, and act in cell division. However, the function of many endogenous siRNAs is unknown. We have found that the ERI-6/7 helicase is required for a subset of endogenous siRNAs present in the nematode *Caenorhabditis elegans*. The ERI-6/7 helicase acts in a pathway together with the Argonaute protein ERGO-1 to produce two types of siRNAs: a primary class of 26 nucleotides in length present in oocytes and embryos, and a class of 22 nucleotide siRNAs present in later stages of development. These siRNAs correspond to only about one hundred genes. Interestingly, we found that these genes fall into groups of genes that contain nearly identical DNA sequences. The sequences of these genes are not conserved in other organisms, not even in related nematodes. These results point to a potential function of these endogenous siRNAs: silencing of recently acquired, duplicated genes. Our work demonstrates a new role of small RNAs, different from known functions in transposon silencing and regulation of gene expression.

## Introduction

Small RNA pathways regulate gene expression, chromatin structure and defense against invading elements such as transposons and viruses. Three general classes of small RNAs can be distinguished in animals: *piwi*-interacting RNAs (piRNAs), microRNAs (miRNAs) and small interfering RNAs (siRNAs). siRNAs exist in two classes: exogenous siRNAs that are derived from an exogenously administered dsRNA and endogenous siRNAs that are naturally generated within cells without administration of a dsRNA. Endogenous siRNAs were first discovered in plants and *C. elegans*, but have since been identified in flies and mouse oocytes [1]–[7]. In addition to regulating gene expression, endogenous siRNAs silence transposable elements and act in chromosome segregation [8]–[10]. Endogenous siRNAs in flies and mice are derived from dsRNA hairpin precursors, from dsRNA generated upon convergent transcription, or from antisense transcription of pseudogenes. What triggers endogenous siRNA formation in *C. elegans* is not as well understood but small RNA deep sequencing experiments have shown that about half of all genes produce endogenous siRNAs suggesting that this regulatory axis controls a wide range of gene activities [10]–[12].

Primary siRNA biogenesis in the exogenous RNAi pathway in *C. elegans* and many other organisms involves enzymatic cleavage by the RNAseIII enzyme Dicer of a longer dsRNA intermediate [13], [14], however, only a subset of endogenous siRNAs requires Dicer (*dcr-1* in *C. elegans*) [12]. siRNAs are incorporated into effector complexes, comprised of an Argonaute protein and accessory factors, which direct silencing of complementary RNAs and in certain species, such as *C. elegans*, recruit RNA-dependent RNA polymerases (RdRPs) to the target resulting in siRNA amplification [15], [16]. In most animals, there are two sub-families of Argonaute proteins, PIWI and Argonaute, that interact with specific classes of small RNAs. In *C. elegans*, there is an additional sub-family of Argonautes that is worm-specific and includes eighteen members. All small RNAs act sequence-specifically through base pairing with their target mRNA, but the outcome of the small RNA:target interaction can vary from suppression of transcription to mRNA degradation or translational repression and this is likely governed in part by the specific Argonaute cofactor.

The *C. elegans* small RNA repertoire includes a large collection of endogenous siRNAs that can be classified by the specific Argonaute they associate with, the length of the small RNA, chemical modifications and the 5′ nucleotide. These include the CSR-1-associated 22G siRNAs (22 nt long with a 5′G) [9], [10], WAGO-associated 22G siRNAs [12] as well as the ERGO-1-associated 26G siRNAs (26 nt long with a 5′G) and ALG-3/4-associated 26G siRNAs [17]–[21] that act upstream of some WAGO-associated 22G siRNAs. Whereas CSR-1-associated siRNAs function in chromosome segregation during meiosis and mitosis, the specific functions of the other three classes of siRNAs are not as clear.

Genetic, molecular and biochemical analyses have identified several genes and proteins involved in endogenous siRNA formation and function. The 26G siRNAs and the corresponding downstream 22G siRNAs, collectively called the ‘ERI’ class of siRNAs, all depend on a protein complex that includes the 3′-5′ exonuclease ERI-1, the RdRP RRF-3, the endonuclease DCR-1/ERI-4, and the dsRNA binding protein RDE-4 [22]–[24]. A subset of ERI class endogenous siRNAs, found in oocytes and embryos, associates with the Argonaute ERGO-1, whereas a sperm-specific class associates with the Argonautes ALG-3 and ALG-4 [18]–[20]. The biogenesis of the downstream, secondary 22G endogenous siRNAs may be mediated by the RdRPs RRF-1 and EGO-1, in conjunction with the helicase DRH-3 [19]–[21]. The 22G siRNAs are incorporated into complexes with one or more of twelve partially redundant worm-specific Argonautes, the WAGOs, including NRDE-3, an Argonaute that directs cotranscriptional gene silencing in the nucleus [25], [26].

ERI-6/7 is a Superfamily I helicase homologous to Mov10 and Mov10-like1 in mice which also act in small RNA mediated gene silencing [27], [28]. The *eri-6/7* mRNA is expressed by *trans*-splicing of the pre-mRNAs of the *eri-6* and *eri-7* genes [29]. Like *eri-1*, *eri-6/7* was identified as a negative regulator of exogenous RNAi, *i.e.* mutants of *eri-6/7* display an enhanced RNAi (Eri) phenotype upon exposure to exogenous dsRNA [29], a phenotype also displayed by *ergo-1*, *eri-4(dcr-1)* and *rrf-3* mutants. To characterize the role of *eri-6/7* in endogenous siRNA pathways, we compared the small RNA profiles of adult and embryo staged *eri-6/7* mutants as well as embryo staged *ergo-1* and *eri-1* mutants to wild type *C. elegans*. Endogenous 26 nt and 22 nt siRNAs corresponding to about one hundred target genes were missing in *eri-6/7* mutants whereas the thousands of other endogenous siRNAs were normally produced. The corresponding mRNA levels of these target genes tested were dramatically up-regulated in the *eri-6/7* mutant, showing that the missing endogenous siRNAs mediate the silencing of these target genes. The *eri-6/7* targets comprise mostly non-conserved genes and pseudogenes and fall into groups with extensive nucleotide sequence homology, indicative of gene duplications. The poor conservation of these genes suggests they may be newly acquired genes. Thus, the results suggest that one function of endogenous RNAi pathways is to silence one or more members of recently expanded gene families, possibly providing selective pressure for one paralog over others and accelerating divergence to avoid silencing. Like *eri-1* and *ergo-1* [18], [19], *eri-6/7* is required for the formation or stability of 26G primary siRNA in embryos and 22G secondary siRNAs derived from 26G siRNA targets in adults. Thus, *eri-6/7*, in collaboration with other ERI class genes, initiates an siRNA cascade of these recently duplicated genes in oocytes and embryos that persists throughout development. Surprisingly, although ERI class siRNAs are Dicer-dependent, the siRNA duplex precursor lacks the canonical features of a Dicer product. Instead of containing the canonical 2 nt 3′ overhangs on each siRNA strand, the 26G siRNA strand has a 3 nt 3′ overhang and an ∼4 nt 5′ overhang. In *ergo-1* mutants, the levels of 26G siRNA passenger strands are elevated relative to wild type, suggesting that ERGO-1 mediates passenger strand removal through endonucleolytic cleavage, analogous to the function of other siRNA-associated Argonautes, such as RDE-1 acting in exogenous RNAi in worms and Ago2 in flies [16], [30].

## Results

### *eri-6/7* is essential for specific classes of endogenous siRNAs

**Figure 1:**
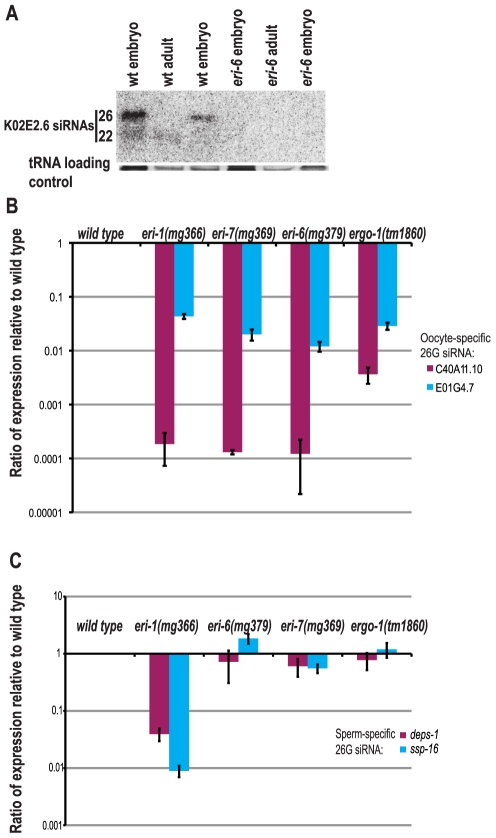
*eri-6/7* is required for the accumulation of distinct classes of endogenous siRNAs. (A) Northern blot analysis of endogenous siRNAs derived from K02E2.6. tRNA is shown as a loading control. (B) Ratio of two oocyte/embryo-enriched 26G siRNAs in *eri* mutant embryos as determined by qRT-PCR (wild type = 1.0). (C) Ratio of two sperm-enriched 26G siRNAs in *eri* mutant L4/young adult animals as determined by qRT-PCR (wild type = 1.0).

The ERI-6/7 helicase was identified as a negative regulator of exogenous RNAi from a genetic screen for mutants that display enhanced RNAi efficacy for exogenously administered dsRNAs corresponding to particular *C. elegans* genes [29]. To understand any possible roles for ERI-6/7 in natural silencing pathways, we analyzed embryo and adult RNA for the presence of endogenous siRNAs by Northern blotting (Figure 1A). We found that two endogenous siRNAs from the gene K02E2.6 are depleted in *eri-6(mg379)* embryos and adults. The longer species is relatively abundant in wild type embryos while absent in adults, as seen for the oocyte/embryo-specific siRNAs that associate with the Argonaute ERGO-1 [19], whereas the shorter species is relatively more abundant in adults. These siRNAs are so-called 26G and 22G siRNAs that depend on ERGO-1, the exonuclease ERI-1 and several other ERI factors [18]–[21]. Using quantitative RT-PCR we specifically assayed for two more oocyte/embryo-specific 26G siRNAs in embryos and also for two sperm-specific 26G siRNAs in young adult hermaphrodites. In embryos, the 26G oocyte-specific small RNAs (ERGO-1 class) were reduced in all *eri* mutants (Figure 1B), whereas the sperm-specific 26G endo-siRNAs (ALG-3/4 class) were unaffected in *eri-6/7* mutants and *ergo-1*, but greatly reduced in *eri-1* (Figure 1C).

**Figure 2:**
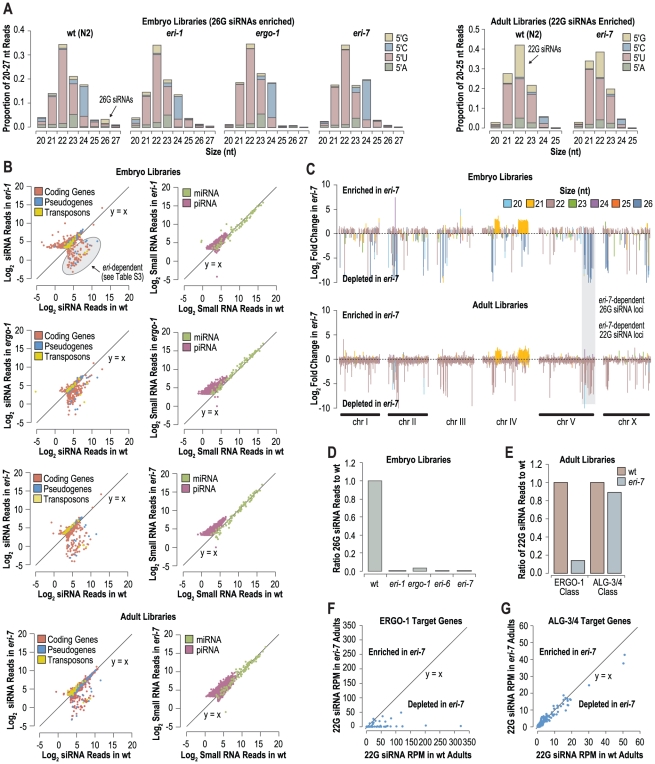
*eri-6/7* is required for accumulation of 26G siRNA in embryos and for a subset of 22G siRNAs in adults. (A) Small RNA length and 5′ nucleotide distribution in wild type and *eri* class mutants. (B) Scatter plots displaying each feature indicated as the number of siRNA reads in wild type versus *eri* class mutants. (C) Ratio of small RNA reads in *eri-7* to wild type embryos and adults after log_2_ transformation. Total small RNA reads within 5 kb interval were plotted across each chromosome in 1 kb increments. Bars are color coded according to the most abundant size class of small RNAs within each interval. (D) Ratio of 26G siRNA reads in *eri* class mutants to wild type embryos (wild type = 1.0). (E) Ratio of ERGO-1 and ALG-3/4 class 22G siRNA reads in *eri-7* mutants to wild type adults (wild type = 1.0). (F) Scatter plot displaying siRNA reads derived from ERGO-1 target genes in wild type versus *eri-7* mutant adults. (G) Scatter plot displaying siRNA reads derived from ALG-3/4 target genes in wild type versus *eri-7* mutant adults.

To more comprehensively assess the requirement for *eri-6/7* in endogenous RNA silencing pathways, small RNA cDNA amplicons were prepared from both embryo and adult staged *eri-7* mutant and wild type *C. elegans* and subjected to deep sequencing. Additionally, small RNA libraries from embryo staged *eri-6*, *eri-1* and *ergo-1* mutants were prepared and sequenced in parallel (Table S1). In adult *eri-7* mutants the small RNA size and 5′ nt distribution was similar to that of wild type, although there was a modest reduction in 22G small RNAs (Figure 2A). In embryos, 26G small RNAs were largely depleted in *eri-7* and *eri-6*, as well as in *eri-1* and *ergo-1* mutants (Figure 2A, Figure S1A). Together with the quantitative RT-PCR data, these data are suggestive of a role for *eri-6/7* in the ERGO-1 endogenous RNAi pathway that generates 26G siRNAs in embryos and 22G siRNAs in adults [19].

To determine if classes of small RNAs other than endogenous siRNAs depend on *eri-6/7*, the numbers of small RNA reads, normalized to library size, corresponding to miRNA genes and piRNA loci, in wild type versus *eri-7*, *eri-6*, *eri-1* and *ergo-1*, were analyzed. Individual miRNAs were not substantially affected in *eri-7*, *eri-6*, *eri-1*, or *ergo-1* (Figure 2B, Figure S1C). piRNAs (also called 21U RNAs in *C. elegans*) appeared slightly upregulated, especially in embryo libraries (Figure 2B and 2C, Figure S1D). Possibly, the lack of 26G siRNAs in embryos has modest effects on embryonic development, which could affect the relative abundance of piRNAs that are known to be more abundant in younger embryos than older embryos [31].

Over half of the genes in *C. elegans* produce endogenous siRNAs through multiple different pathways [11], [12]. We analyzed which loci produce *eri-6/7*-dependent siRNAs. First small RNA reads for each coding gene, pseudogene and transposon were plotted as a function of library size normalized reads in wild type versus *eri-7*, *eri-6*, *eri-1* and *ergo-1* mutants (embryos) and in wild type versus *eri-7* (adults) (Figure 2B, Figure S1B). Using an arbitrary read threshold of 10 reads per million total reads (RPM) for wild type small RNA libraries, ∼80 features, primarily annotated coding genes and pseudogenes, were depleted of siRNA reads by ≥67% in both *eri-7* and *eri-1* mutant embryos, relative to wild type (Figure 2B, Figure S1B, Table S3). A similar, albeit more modest, reduction in siRNA reads was observed in *ergo-1* mutants (Figure 2B, Table S3). A partially overlapping set of features was also depleted of siRNAs in adult *eri-7* mutants (Figure 2B). To account for intergenic and other non-annotated loci, we plotted the ratios of small RNA reads in *eri-7*, *eri-6*, *eri-1* and *ergo-1* mutants to wild type in 5 kb windows along 1 kb increments across each chromosome (Figure 2C, Figure S1D). Loci depleted of siRNAs in *eri-6*, *eri-7*, *eri-1*, and *ergo-1* mutants largely overlapped and tended to derive from the more gene-poor arms of the chromosomes, as was observed for *ergo-1* by Vasale *et al.* [19] (Figure 2C, Figure S1D). The majority of loci depleted of siRNAs correspond to annotated coding genes and pseudogenes and predominantly yield 26 nt small RNAs in wild type embryos (Figure 2C, Figure S1D). A total of 1160 individual 26G siRNAs were identified that passed a read threshold of 1 RPM and were depleted by ≥67% in both *eri-7* and *eri-1* mutants, relative to wild type (Table S2). In total, 26G siRNAs were depleted by >99% in *eri-6*, *eri-7* and *eri-1* mutant embryos and by ∼96% in *ergo-1* mutant embryos, relative to wild type (Figure 2D). In adults, the loci depleted of small RNAs in *eri-7* mutants largely overlapped with those in embryos, but were predominantly of the 22 nt size class (Figure 2C) and therefore likely correspond to the secondary 22G siRNAs that are thought to be downstream of 26G siRNAs [19], [21].

22G siRNAs derived from ERGO-1 class 26G siRNA targets are biochemically indistinguishable those derived from ALG-3/4 class 26G siRNA targets. Although our qRT-PCR results suggested that *eri-6/7* is not required for ALG-3/4 class 26G siRNA accumulation, we nonetheless assessed the requirement of *eri-6/7* for 22G siRNAs derived from both ERGO-1 and ALG-3/4 class 26G siRNA targets using published ERGO-1 and ALG-3/4 target datasets [18]–[20]. Consistent with a requirement for *eri-6/7* specifically in the ERGO-1 class 26G siRNA pathway, 22G siRNAs derived from ALG-3/4 class 26G siRNA targets were unaffected in *eri-7* mutant adults, whereas 22G siRNAs corresponding to ERGO-1 class 26G siRNA targets were depleted by ∼90% (Figure 2E). Similarly, 22G siRNA reads from individual ERGO-1 class 26G siRNA targets were largely depleted and 22G siRNA reads from individual ALG-3/4 class 26G siRNA targets were unaffected in *eri-7* mutant adults (Figure 2F–2G). ERGO-1 class 22G siRNAs are relatively more abundant in adults versus embryos, confirming the observations made by Northern blot analysis (Figure 1A, Figure S3). The majority of 22G siRNAs, including those that associate with the Argonaute CSR-1 to direct chromosome segregation, are not dependent on either class of 26G siRNAs. These non-26G-dependent 22G siRNAs were not depleted in *eri-7* mutant adults, relative to wild type (Figure S2). Thus, *eri-6/7* is specifically required for ERGO-1 class 26G siRNA formation in oocytes and embryos and, possibly indirectly, for accumulation of the secondary 22G siRNAs present in the adult.

### *eri* target loci are related in sequence

**Figure 3:**
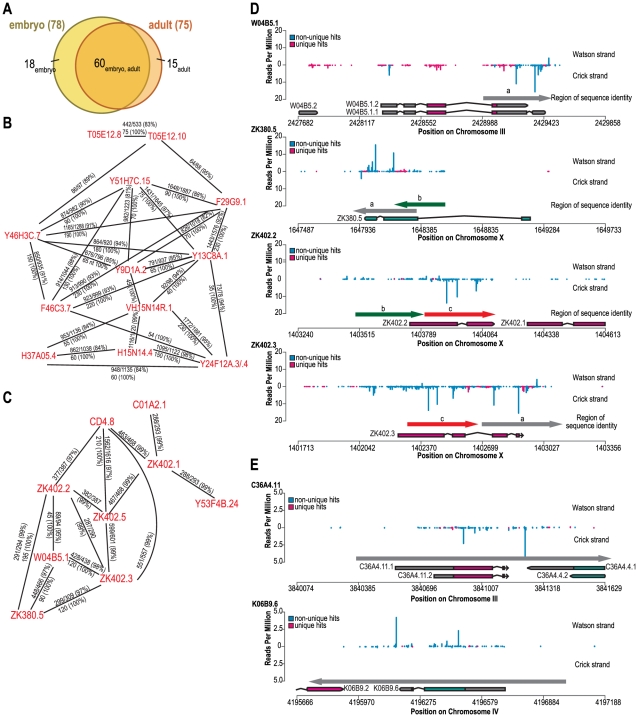
Genes targeted by *eri-6/7*-dependent endogenous siRNAs share regions of high sequence identity. (A) Overlap between genes targeted by *eri-6/7*-dependent siRNAs identified by deep sequencing of embryo small RNA and of adult small RNA. (B, C) Two groups of target genes that share sequence identity. Indicated are stretches with high identity and stretches of 100% identity. (D) Target genes show sequence identity in regions with siRNAs. siRNA reads in wild type embryo libraries were plotted against four target gene regions. Unique siRNAs and non-unique siRNAs are color-coded. Gene structures of different splice variants are shown with genes on the Watson strand pointing to the right, genes on the Crick strand pointing to the left. Regions of high sequence identity are indicated by arrows, color-coded and marked by a letter. (E) Homology between the adult *eri-6/7*-dependent siRNA target genes C36A4.11 and K06B9.6. Unique and non-unique siRNAs are color-coded. The grey arrow indicates the region of identity.

From our deep sequencing datasets we identified 78 and 75 annotated coding genes, pseudogenes, and transposons that yielded ≥10 RPM and were depleted by >67% in *eri-7* mutant embryos and adults, respectively, relative to wild type (Table S3). Of these, 60 are depleted of siRNAs in both embryo and adult *eri-7* mutants (Figure 3A). The endogenous siRNAs derived from genes and pseudogenes did not always appear antisense to the predicted exons as annotated in Wormbase. However comparison to recent modENCODE data [32] shows that for these loci intron/exon structures are either incorrectly annotated or not annotated in Wormbase.

Many *eri-6/7*-dependent endogenous siRNAs do not match a unique sequence in the genome, as initially noticed by Vasale *et al.* [19] for *ergo-1* target genes. To analyze the extent of homology between the *eri-6/7* target genes, the sequences of all annotated coding genes and pseudogenes, including 0.5 kb of flanking sequence on either side, were retrieved and aligned using the blastn and discontinuous megablast algorithms. Surprisingly, over two-thirds of the genes targeted by *eri-6/7*-dependent siRNAs share >82% total nt identity to one or more of the other 78 *eri-6/7* target genes as well as stretches of ≥27 nt with 100% identity (Figure 3B and 3C, Table S3, Figure S4). In contrast, within a set of randomly selected genes of the same combined sequence length, only 4% share this degree of sequence identity. Whereas some target genes represent pairs of homologous genes (Figure 3E), many genes form large clusters that share sequence homology (Figure 3B and 3C), although within these clusters, not all genes show homology to the same sequence (Figure 3D).

Fourteen genes not only share sequence homology but are also in close proximity (within 5 kb) of each other (Table S3, Figure S4). Some of these genes are adjacent to each other and are likely the result of recent gene duplications [33] (e.g.W04B5.6 and E02H9.6). However, seven genes are in close proximity but not homologous, suggesting that the endogenous siRNA biogenesis machinery has a preference for certain genomic regions, possibly determined by chromatin state.

There are common features that may route these genes into the *eri-6/7*-dependent endogenous siRNA pathway. The protein sequences of target genes with *eri-6/7*-dependent siRNAs are poorly conserved. More than half of the *eri-6/7* target genes are not conserved between *C. elegans* and the related nematode *C. briggsae*, which is about five times the genome average of 11% of genes that are not shared between the two species [34]. The 34 *eri-6/7* target genes with a detectable homolog in *C. briggsae* rank among the least conserved between the two species; these genes had a much higher median E-value, 5.9E-07 as compared to a median of 5.8E-114 for all *C. elegans* gene products with a *C. briggsae* homolog (Table S4). The gene structure of the *eri-6/7*-dependent siRNA target genes also deviates from the typical *C. elegans* gene; the genes are shorter (1.3 kb average versus 2.8 kb) than the average protein coding gene in *C. elegans* and contain fewer exons (median of 3 versus 5 for all *C. elegans* genes) (Table S5). The lack of conservation even in other nematodes, and lack of other indications of function for the majority of these genes suggest that they were recently acquired by *C. elegans* and that some of these genes may not produce functional proteins, *i.e.* they are pseudogenes. Indeed, in an analysis of a large scale proteomics dataset [35] we identified peptides from only 16% of *eri-6/7* target genes, compared to 54% for all annotated coding genes (Table S6). The fact that many endogenous siRNAs match gene sequences repeated in the genome, suggests that the *eri-6/7* pathway targets duplicated genome segments. Gene duplications occur at a relatively high frequency in *C. elegans* [36], and tend to produce partial or chimeric gene duplicates [33]. Sixteen *eri-6/7* target genes are among the 516 most recently duplicated genes present in the whole genome based on low synonymous substitution numbers (K_s_<0.1) between duplicated genes [36], a seven-fold enrichment over random expectation.

The physiological role of the *eri-6/7* pathway remains undefined. Whereas *eri-1* mutants display temperature-sensitive sterility due to defects in sperm morphology related to the ALG-3/4 endogenous siRNA pathway, *eri-6/7* and *ergo-1* mutants show no obvious phenotypes. However, brood size analysis at elevated temperatures indicates that down-regulation of *eri-6/7* target genes may be required for optimal fecundity at higher temperatures (Table S7). Many of the *eri-6/7* target genes lack indication of function; of 50 *eri-6/7* target genes tested by the *C. elegans* community for gene inactivation phenotypes (as annotated in Wormbase), only three produce RNAi-induced phenotypes (6%), whereas from the whole genome, 5,852 out of 20,808 (28%) gene inactivations induce phenotypes. However, several of the target genes are homologous to nucleic acid modifying enzymes, such as the gene F55C9.3 (PAZ domain), the genes F39E9.7 and F39E9.10 (dsRNA binding domain) as well as several predicted helicase genes. Possibly, upregulation of these target genes regulated by *eri-6/7* contributes to the enhanced RNAi phenotype of the *eri-6/7* mutants.

### *eri-6/7*-dependent siRNAs feed into in the NRDE co-transcriptional silencing pathway

**Figure 4:**
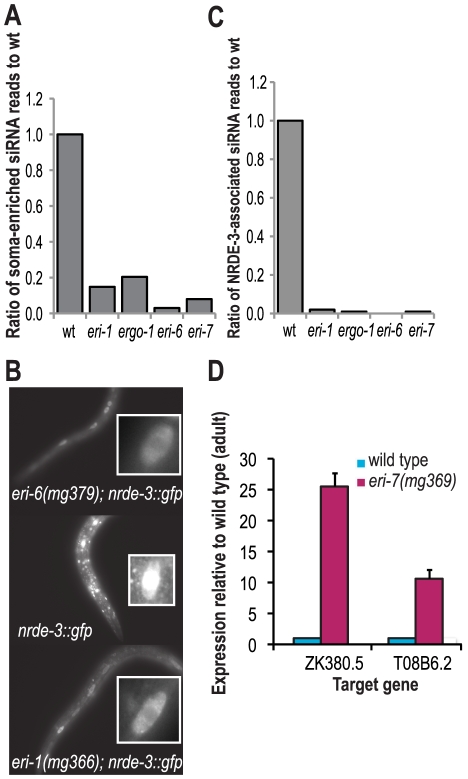
*eri-6/7*-dependent siRNAs associate with NRDE-3 to direct cotranscriptional gene silencing in the nucleus. (A) Ratio of soma-enriched siRNAs in *eri* class mutants to wild adults (wild type = 1.0). (B) RDE-3 localization in wild type and *eri-6* mutants. The insets show single hypodermal cells with either mostly cytoplasmic (*eri-1* and *eri-6* mutants) or nuclear (in wild type) GFP::NRDE-3 expression. (C) Ratio of NRDE-3-associated siRNA reads in *eri* class mutants to wild type adults (wild type = 0). (D) Ratio of mRNA levels of two genes that yield *eri-6/7*-dependent siRNAs in *eri-7* mutants to wild type adults, as determined by qRT-PCR (wild type = 1.0).

*eri-6/7*-dependent siRNAs exist as two classes: the primary 26G siRNAs found in oocytes and embryos and secondary 22G siRNAs that predominate in post-embryonic stages of development. By analysis of genes shown to be enriched for siRNAs in the soma or germline [12] we determined that the *eri-6/7*-dependent secondary endogenous siRNAs are mostly soma-enriched (Figure 4A).

Four Argonaute proteins are known to act somatically [26], including SAGO-1, SAGO-2 and NRDE-3, and could thus potentially interact with *eri-6/7*-dependent 22G siRNAs. By Northern blot analysis, an *ergo-1*-dependent 22G siRNA was found to be dependent on *sago-1* and *sago-2* [19]. NRDE-3, an Argonaute protein that acts in co-transcriptional silencing of endogenous siRNA targets in the nucleus [25], [38], associates with *eri-1*-dependent 20–22 nt siRNAs that are required for NRDE-3 localization to the nucleus. *nrde-3* is also partially required for an exogenous RNAi response: the enhanced RNAi phenotype of *eri-1* mutants in response to dsRNA triggers that target nuclear RNAs is dependent on *nrde-3*, but non-nuclear RNAi responses in *eri-1* mutants are independent of *nrde-3* [25].

To examine if *eri-6/7* acts in the NRDE-3 pathway, double mutants of *eri-6/7* with *nrde-3* were assayed for enhanced RNAi phenotypes (Eri) and transgene silencing phenotypes. *nrde-3* is required for the enhanced RNAi phenotype of *eri-6/7* mutants in response to *dpy-13(RNAi)*, similar to *eri-1* mutants (Table S8). Like other *eri* mutants such as *rrf-3* [39], *eri-6/7* mutants display silencing of repetitive transgenes (Table S8). *nrde-3* mutants display weak silencing of repetitive transgenes, whereas *nrde-3;eri-6* double mutants do not display transgene silencing (Table S8). These data suggest the existence of multiple, possibly competing, transgene silencing pathways regulated by *nrde-3* and *eri-6/7*.

The nuclear function of *eri-6/7*-dependent siRNAs was further examined by NRDE-3 localization analysis in *eri-6/7* mutants at post-embryonic stages. Like in *eri-1* mutants, NRDE-3 fails to localize to the nucleus in *eri-6/7* mutants, suggesting that a subset or all 22G endogenous siRNAs dependent on *eri-6/7* direct NRDE-3 to the nucleus to mediate co-transcriptional gene silencing (Figure 4B). Indeed, analysis of the NRDE-3-associated siRNAs [25] indicates that these siRNAs are depleted to 1% or less of the wild type values in *eri-6/7* mutants (Figure 4C). Thus, NRDE-3 requires *eri-6/7*-dependent siRNAs for localization to the nucleus where it mediates co-transcriptional gene silencing [25].

To assess the effects of loss of *eri-6/7* on target mRNA levels, we performed quantitative RT-PCR on several *eri-6/7* target genes, including ZK380.5 and T08B6.2. We saw 10–25 fold increases in target mRNA levels in *eri-6* mutant young adult samples as compared to wild type (Figure 4D, Figure S7) similar to what has been seen in other *eri* pathway mutants [18], [19], [21], [23]. mRNA levels of *eri-6/7* target genes are also increased in the *nrde-3* mutant as compared to wild type (Figure S7 and [25]).

In conclusion, *eri-6/7* mutants are depleted of NRDE-3-associated siRNAs and show a disruption of NRDE-3 localization to the nucleus. In post-embryonic stages, *eri-6/7*-dependent siRNAs silence their targets leading to reduced mRNA levels, likely through co-transcriptional silencing via the NRDE pathway in the nucleus, although additional modes of silencing may exist. In oocytes and embryos, *eri-6/7*-dependent primary (26G) siRNA biogenesis likely contributes to target silencing by Dicer-mediated cleavage of the target mRNA (as discussed below).

### Features of 26G siRNA duplexes suggest non-canonical Dicer processing

**Figure 5:**
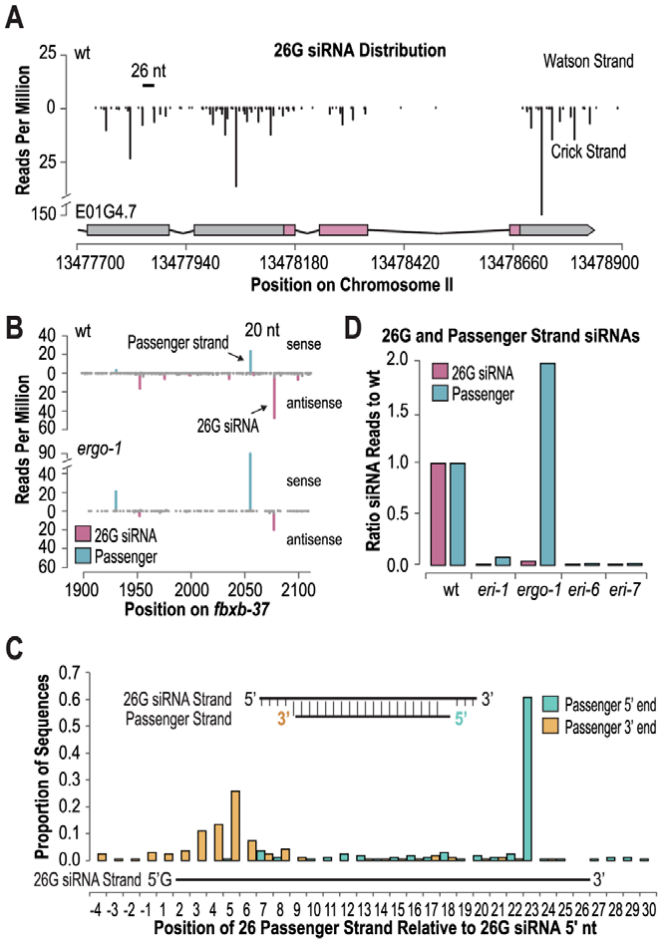
26G siRNAs are derived from non-canonical siRNA duplexes. (A) 26G siRNA distribution (indicated are 5′ positions) across the E01G4.7 locus. (B) siRNA distribution across the *fbxb-37* locus. (C) The proportions of 5′ and 3′ ends of the most abundant sequences overlapping and antisense to each 26G siRNA sequence are shown. Inset displays the consensus 26G siRNA duplex. (D) Ratio of 26G siRNA passenger strand reads in *eri* mutants to wild type (wild type = 1.0).

The mechanism of 26G siRNA formation is not well understood. In *C. elegans*, exogenous siRNAs are processed from a long dsRNA via processive Dicer activity generating ∼23 nt siRNAs starting at one end of the dsRNA [14]. Similarly, in plants, (endogenous) trans-acting siRNAs (tasiRNAs) are processed by Dicer in sequential 21 nt increments in phase with a miRNA-guided cleavage site [40]. We observed that 26G siRNAs are also produced in regular but variable intervals, typically ranging between 23–29 nt (Figure 5A, Figure S5), with an siRNA length of invariably 26 nucleotides. The pattern of 26G siRNA distribution does not point to a specific initiation site, except for a preference for the 5′ end to start opposite a cytosine on the complementary mRNA, nor is it consistent across a given transcript. It is likely that the regular patterning of 26G siRNAs results from a combination of a processive RdRP activity and endonucleolytic activity by Dicer (DCR-1). In support of this, DCR-1 is found in a complex with the RdRP RRF-3, and both these factors are required for 26G siRNA formation [22]–[24], [41].

During exogenous siRNA formation, the functional guide strand of an siRNA duplex intermediate is liberated from a passenger strand [16], [30], [42]–[44]. Given the requirement for DCR-1 in 26G endogenous siRNA formation, we predicted that 26G siRNAs are also liberated from a duplex intermediate. To identify potential 26G siRNA passenger strands, we searched our small RNA libraries for sequences that at least partially overlapped and were antisense to each 26G siRNA. For 1100 of the 1160 26G siRNAs examined, we were able to identify a sequence that met our criteria. Canonical Dicer products are 21–24 nt long and have 2 nt overhangs at each 3′ end of the small RNA duplex, however, the most dominant sequence antisense to each 26G siRNA was ∼19 nt long and inset by 3 nt from the 3′ end and ∼2–4 nt from the 5′ end of the 26G siRNA (Figure 5B, 5C, Table S9). The ratio of passenger strand reads to corresponding 26G siRNA reads after applying a 1 RPM threshold ranged from 0 to 6.4 with a median ratio of ∼0.05 (Table S8). Similar to the 26G siRNA strand, the passenger strand was depleted in *eri-7*, *eri-6* and *eri-1* mutants, relative to wild type, suggesting that these factors are upstream of 26G siRNA duplex formation (Figure 5D). In contrast, in *ergo-1* mutants, the passenger strand was elevated by ∼2 fold, relative to wild type (Figure 5B and 5D). The observation that the passenger strand is stabilized in *ergo-1* mutants suggests that, similar to RDE-1 in worms and AGO2 in flies, ERGO-1 cleaves the passenger strand to liberate it from the 26G siRNA [16]. Indeed, ERGO-1 is one of only a few *C. elegans* Argonautes that contains the conserved RNaseH residues required for slicer activity. From a genetic screen for enhanced exogenous RNAi mutants [29], [45], we isolated an *ergo-1* mutant, *ergo-1(mg394)*, that has a amino acid substitution at position 1072 in the presumable catalytic pocket defined by the D(852)D(930)H(1070) amino acids required for slicer function. This mutant has a enhanced RNAi phenotype indistinguishable from the presumable null mutant, the deletion mutant *ergo-1(tm1860)*. This suggests that slicer function is required for wild type ERGO-1 activity.

The features of 26G siRNAs and their passenger strands suggest a novel non-canonical mechanism for Dicer activity on 26G siRNA precursors, possibly facilitated by another ribonuclease in the pathway, ERI-1.

## Discussion

The ERI-6/7 helicase is a negative regulator of exogenous RNAi and as we have shown here, is required for a particular suite of endogenous siRNAs in what is now emerging as a multidimensional set of endogenous RNAi pathways. ERI-6/7 is, like the Argonaute ERGO-1, required for the generation and/or stability of two classes of siRNAs, oocyte- and embryo-specific 26G siRNAs and the later generated somatic secondary 22G siRNAs corresponding to the same loci. These 22G siRNAs reduce target mRNA levels, similar to secondary siRNAs in the exogenous RNAi pathway. Our analysis of the ERGO-1/ERI-6/7 pathway has two major surprises: first, that this pathway targets a relatively small number of loci in the genome, a set of duplicated genes with extensive nucleic acid homology. This points to a dedicated surveillance pathway for such gene duplications. Because so many of the components of the ERGO-1/ERI-6/7 pathway are conserved across phylogeny, this duplicated gene silencing pathway is likely to be general. Secondly, the detailed deep sequencing analysis of *eri-6/7*-dependent small RNA revealed the presence of a passenger strand and (imperfect) phasing between 26G siRNAs, adding to our understanding of the mechanism of endogenous siRNA biogenesis and the role of the Argonaute ERGO-1.

The set of *eri-6/7* target genes revealed by our deep sequencing analysis consists of pairs or larger groups of genes that share extensive DNA sequence homology, have a small number of introns and are poorly conserved, even in other *Caenorhabditae*. The poor conservation and few introns support the model that these genes have recently been acquired by *C. elegans*, perhaps via horizontal gene transfer, for example, from viruses. RNAi pathways have been implicated in antiviral defense, and the ERGO-1/ERI-6/7 pathway may constitute elements of such a viral surveillance pathway. Our data suggest that viral surveillance extends beyond the initial infection. Newly acquired viral genomes may tend to integrate at multiple loci so that extensive nucleotide sequence homology between disparate loci may be a signature of such genes and continue to be silenced by the *eri-6/7* small RNA pathway. Alternatively, these duplicated genes may be novel DNA transposons. We did not find evidence of target site duplication at the boundaries of the homologous sequences, nor did we find terminal inverted repeats. If these are transposons, they may no longer are be active, even in *eri* mutants; although the level of sequence identity is high, it is not as high as of active DNA transposons in *C. elegans* [46]. Also, we have not found evidence of mutator activity in *eri* mutants.

The targeting of these genes with extensive nucleotide homology suggests that the ERI-6/7 helicase, the ERGO-1 Argonaute protein and other ERI proteins must specifically generate or load siRNAs from the duplicated segments of such gene pairs. To achieve this specificity for duplicated genes, transcripts of every gene may be compared to every other gene and extensive but not perfect nucleotide homology over a distance of hundreds of nucleotides may be assessed by this system. Such a system would need to detect the distinct genome location or the small level of nucleotide sequence divergence that would distinguish surveillance of another transcript from the same gene from a transcript emerging from a duplicated distinct gene.

The most obvious phenotype of the *eri-6/7* or *ergo-1* null mutations is enhanced response to exogenous RNAi. Our data shows that *eri-6/7* mutants have a reduced brood size. The reduced fitness could be attributed to over-expression of specific target genes that act in specific pathways or by more systemic effects induced by a general accumulation of unwanted RNAs. The lack of functional annotation for the majority of *eri-6/7* target genes suggests some of these genes may not be functional, though about half of the target genes are weakly conserved in *C. briggsae*. Thus, the surveillance of these duplicated genes does not appear to subserve any key function for development or survival in the lab. However, this surveillance program uses sufficient *C. elegans* small RNA machinery that when the *eri-6/7* system is disabled, the ability of the animal to respond to exogenous double stranded RNA is enhanced. The small RNA demands of this pathway point to its importance to the organism.

Our data provides insight into the structure of the double-stranded intermediate in 26G siRNA generation by the identification of the passenger strand and the first large scale sequence analysis of the passenger strand in a slicer-defective Argonaute mutant. An obvious candidate for duplex siRNA generation is Dicer/DCR-1, which was shown to be required for 26G siRNA biogenesis [18], [19], [23], [24]; A helicase domain mutation *(dcr-1/eri-4(mg375))* in DCR-1 specifically abolishes endogenous siRNAs but not microRNAs [21], [23], [24], indicating that the requirement for Dicer is unlikely an indirect effect via a microRNA target gene that acts in 26G siRNA biogenesis. However, the 26G siRNA duplexes are not canonical Dicer products in terms of the lengths of the antisense and passenger strands, the 5′ overhang, and the strong bias for a G as the 5′ nucleotide. Our observation of variable phasing between the 5′ ends of the 26G siRNAs within genes, suggest there is a processive activity that generates 26G siRNAs. This is most likely the RdRP RRF-3, preferentially using a guanylate as an initiation nucleotide, that in conjuction with an endonuclease, possibly Dicer, generates a 26G siRNA duplex and continues doing so along the mRNA starting at a neighboring cytosine in the mRNA template [17]. The structure of the duplex suggests that it is modified by other enzymes, such as the ERI-1 3′ exonuclease, to produce the 19 nucleotide passenger strand (Figure S6). Our *eri-1* mutant deep sequencing data did not provide evidence for the existence of longer passenger strand precursors; Possibly such precursors are not stable.

*eri-6/7* acts in the same pathway as the Argonaute ERGO-1 but unlike *eri-6/7*, *ergo-1* is not required for the passenger strand opposite of the 26G siRNA. This suggests that *eri-6/7* acts, with *eri-1* and other *eri* genes, in the production of a 26G siRNA duplex precursor, while *ergo-1* acts on the duplex after biogenesis, removing the passenger strand possibly by slicing, similar to the function of another slicing-capable Argonaute RDE-1 in exogenous RNAi in *C. elegans*, and similar to the roles of Argonautes in flies and mammals [16], [30], [42], [43]. Site-directed mutagenesis experiments of the catalytic amino acids DDH are necessary to provide more direct evidence of slicing versus other ways of passenger strand destabilization by ERGO-1. The role of ERGO-1 in passenger strand removal versus siRNA biogenesis could also explain the weaker reduction in siRNAs seen in *ergo-1(tm1860)* mutants versus *eri-1* and *eri-6/7* mutants. Alternative explanations are that the *ergo-1(tm1860)* allele is a partial loss-of-function, although the deletion removes more than one third of the PAZ domain, or that other Argonautes are partially redundant with *ergo-1*.

The molecular function of the ERI-6/7 helicase is unclear. The homologous protein Mov10 in humans associates with Argonaute [47] and the fly homolog Armitage is required for RNA induced silencing complex (RISC) formation [48]. Thus it is possible that ERI-6/7 interacts with ERGO-1 and functions in the assembly of an active effector complex. *eri-6/7* does not act in the sperm-specific 26G siRNA pathway that involves the Argonautes ALG-3/4 in place of ERGO-1; it will be of interest to determine if another helicase functions in 26G endogenous siRNA generation in this pathway.

Vasale *et al.* and Gent *et al.* [19], [21] have proposed a two-step model for siRNA generation in the ERGO-1 pathway. Downstream of 26G siRNAs, 22G siRNAs are generated by RNA-dependent RNA polymerases RRF-1 and EGO-1. Our data suggests that these events are actually spaced in time, with 26G siRNA generation first in the developing embryo and subsequent RdRP-mediated 22G siRNA generation occurring post-embryonically.

mRNA levels of *eri-6/7* target genes are down-regulated in wild type worms compared to *eri* mutants. This is explained in part by routing of the endogenous siRNAs into a nuclear co-transcriptional silencing pathway that involves the Argonaute NRDE-3. *eri-6/7*-dependent siRNAs are also likely to associate with other Argonautes, such as SAGO-1 and SAGO-2, since at least two *eri-6/7*-dependent endogenous siRNAs, assayed by Northern blotting, were shown to associate with SAGO-1 and -2 [26]. How these Argonautes affect target gene expression is unknown, but the lack the catalytic residues required for slicing, suggests that they direct mRNA degradation by some means other than slicing or that they inhibit translation.

In *eri* mutants, that are defective in some endogenous RNAi pathways, the exogenous RNAi pathway is more active. The opposing functions of *eri-6/7* (and other *eri* genes) in exogenous RNAi and endogenous RNAi have been explained by a competition model in which the exogenous RNAi pathway competes with the endogenous RNAi pathway for limiting factors [22], [49]. The *alg-3/-4* double mutant does not show an enhanced RNAi phenotype [18], suggesting that the limiting factors that the exogenous RNAi pathway competes for are only part of the embryo- and soma-specific ERGO-1/ERI-6/7 endogenous siRNA pathway. The observation that overexpression of the SAGO-1 and -2 proteins that interact with secondary siRNAs causes an Eri phenotype and an enhanced accumulation of endogenous siRNAs [26], shows that these SAGOs could be the limiting factors in the exogenous RNAi pathway. It remains possible that one or more target genes regulated by the *eri* genes also act in RNAi pathways. Several target genes encode proteins with potential RNA modifying capability, such as a few helicases, dsRNA binding proteins and a PAZ domain protein.

Whereas in mouse oocytes endogenous siRNAs are formed from antisense pseudogene transcripts, *C. elegans* has RNA-dependent RNA polymerases, in this case possibly RRF-3, that can produce antisense transcripts. How RRF-3 is recruited to target mRNAs to generate antisense siRNAs is unknown. Possibly, short double stranded RNAs of mRNAs base pairing with antisense transcripts generated from homologous genes recruit the RNA-dependent RNA polymerase. Only a few *eri-6/7* target genes have been annotated as pseudogenes, but it remains possible that among the annotated coding genes targeted in *eri-6/7* mutants are also pseudogenes. Thus, similar to the function of some pseudogenes in mouse oocytes, pseudogenes in *C. elegans* may play an important role in endogenous RNAi.

## Materials and Methods

### *C. elegans* growth and handling

All experiments were performed at 20°C unless stated otherwise. For deep sequencing analysis, wild type N2, *eri-6(mg379)* (6× backcrossed), *eri-7(mg369)* (4× backcrossed), *eri-1(mg366)* (5× backcrossed) and *ergo-1(tm1860)* (5× backcrossed) were used. YY174 (NRDE-3p::3xFLAG::GFP::NRDE-3) and YY178 *(eri-1(mg366)*;NRDE-3p::3xFLAG::GFP::NRDE-3) [25] were used in GFP::NRDE-3 sub-cellular localization analyses and crosses to *eri-6(mg379)*. Enhanced RNAi assays and transgene silencing were performed as described previously [29]. WM156 *(nrde-3(tm1115))* was used in enhanced RNAi assays, transgene silencing assays and qRT-PCRs.

### Microscopy

NRDE-3::GFP localization was determined by Normarski and fluorescence imaging. GFP images were taken at 40× with a 100 ms exposure; DIC images were exposed for 59 ms.

### RNA isolation

Total RNA was isolated by dounce homogenization in RNA-Bee (Tel-Test) followed by chloroform extraction and isopropanol precipitation.

### Northern blotting

30 µg of total RNA was run on a 15% polyacrylamide/urea gel, blotted and probed with a Starfire probe detecting an endogenous siRNA corresponding to gene K02E2.6 [22].

### qRT–PCR

For qRT-PCR, total RNA was DNase treated using the TURBO DNA-free kit (Applied Biosystems). cDNA was synthesized using RETROscript (Applied Biosystems) following the vendor's protocol. qPCR was done with CFX96 machine (Bio-Rad) using iQ SYBR Green Supermix (Bio-Rad). Relative mRNA levels were calculated based on the 2^−ΔΔct^ method using the gene Y45F10D.4 for normalization [50]. Three technical replicates were done for each PCR. Primer sequences are listed in Table S10.

### Taqman qRT–PCR

Taqman qRT-PCR and data analysis was carried out as described [18]. For ERGO-1-class and ALG-3/4-class 26G siRNAs, total RNA was extracted from embryos and 52–56 hr post hatching L4/young adult worms, respectively.

### Small RNA sequencing

Total RNA was isolated from worms grown at 20°C for ∼66–72 hrs post synchronization at the L1 stage and harvested as day one gravid adults, or from embryos. 18–28 nt small RNAs were size selected, tobacco acid pyrophosphatase treated to remove 5′ di- and triphosphate groups, ligated to 5′ and 3′ adapters and subjected to RT-PCR, according to the protocol by Gu *et al.* [12]. Small RNA amplicons were sequenced using an Illumina Genome Analyzer.

### Small RNA data analysis

Data analysis was done as described [11]. Briefly, sequences were parsed and mapped to the *C. elegans* genome (Wormbase release WS203) using CASHX (version 2.0) [51]. Small RNA reads from each library were normalized to the total number of mapped reads. The numbers of small RNA reads for small RNA sequences mapping to multiple positions in the genome were divided by the corresponding number of genomic loci. Small RNA reads were classified by genomic feature according to Wormbase annotations (WS203). Genome plots were constructed by plotting total small RNA read counts from 5 kb windows along 1 kb increments of each chromosome. siRNAs were classified using published datasets [11], [12], [18]–[20]. NRDE-3-associated siRNAs were identified by Guang *et al.* [25]. The passenger strand was identified by taking the genomic coordinates of each 26G siRNA, adding 14 nucleotides of flanking sequence at each end and extracting sequences antisense to and within this 54 bp sequence from our wild type embryo small RNA library. Based on results from our analyses, we defined the 26G siRNA passenger strand as the most abundant antisense siRNA inset by 3 nt from the 3′ end of the 26G siRNA (Table S9).

### Target gene analyses

Target gene sequences including 0.5 kb of 5′ and 3′ flanking sequence were obtained from Wormbase. Subsequently, these sequences were analyzed by discontiguous megablast and blastn for sequence identity. The best protein homologs in *C. briggsae* and *H. sapiens* for all *C. elegans* gene products were identified using Wormbase, with an E-value of 0.1 as a cut-off. Target genes that for which the intron/exon predictions are consistent between Wormbase and modENCODE data were analyzed for number of exons and gene length and compared to all genes in the genome. Peptides produced from *eri-6/7* target genes were mined from Schrimpf *et al.* [35].

### Accession numbers

Small RNA Illumina deep sequencing data are available at the Gene Expression Omnibus (GEO) database (accession no. GSE32366).

## Supporting Information

Figure S1 (A) 5′ nucleotide and length analysis of the small RNA of *eri-6* mutant embryos. (B) Scatter plot showing types of features with reduced siRNAs abundance in *eri-6* mutants. (C) Scatter plot showing that miRNA and piRNA levels are unchanged in *eri-6* mutants. (D) Genome plots of siRNAs enriched and depleted in the *eri* mutants plotted against the six *C. elegans* chromosomes.
(EPS)

Figure S2 WAGO and CSR-1 class endogenous siRNA are not dependent on *eri-6/7*. The ratio of endogenous siRNA read numbers in *eri* mutant versus wild type are graphed.
(EPS)

Figure S3 ERGO-1 and ALG-3/4 class 22G siRNAs are reduced in wild type embryos versus wild type adults.
(EPS)

Figure S4 Homology between embryonic *eri-6/7*-dependent siRNA target genes. Indicated are the lengths of regions of high identity and approximate lengths regions of 100% identity. Dashed lines indicate proximity of genes within 5 kb.
(EPS)

Figure S5 Phasing between the 5′ ends of 26G siRNA reads in wild type embryos antisense to *fbxb-37*/T16A1.8. Plotted are the number of reads per million for each 26G siRNA, versus the position of the 5′G nucleotide of the siRNA. Indicated in red is the 5′ nucleotide of the siRNA reads. The spacing between 5′ nucleotides is indicated.
(EPS)

Figure S6 Model of *eri-6/7* and *ergo-1* function in endogenous RNAi.
(EPS)

Figure S7 Ratio of mRNA levels of *eri-6/7* target F55C9.3 in *eri-6*, *nrde-3* and *eri-6;nrde-3* embryos and adults to wild type, as determined by qRT-PCR (wild type = 1.0).
(EPS)

Table S1 Deep sequencing statistics.
(DOC)

Table S2 Sequence, genomic location and abundance of 26G siRNA reads in embryos in *eri* mutants.
(XLSX)

Table S3 Target genes of *eri-6/7*-dependent siRNAs. Listed are feature targeted, genomic position, abundance, relative abundance to wild type, other target genes with high sequence identity.
(XLS)

Table S4 Gene products *eri-6/7*-dependent siRNA target genes are relatively poorly conserved in the related nematode *C. briggsae* and in *H. sapiens*.
(DOC)

Table S5 *eri-6/7*-dependent siRNA target genes consist of relatively few exons and are short.
(DOC)

Table S6 Proteomics analysis of mixed stage *C. elegans* [35] shows that few *eri-6/7* target genes produce proteins.
(DOC)

Table S7 Brood size of *eri-6/7* mutants.
(DOCX)

Table S8 Enhanced RNAi and transgene silencing phenotypes of *eri-6* and *eri-6;nrde-3* double mutants.
(DOC)

Table S9 Passenger strand analysis. Position, sequence and abundance of passenger strands and their corresponding 26G siRNAs.
(XLSX)

Table S10 Primers used in this study.
(DOC)
