# Supplementary material for: The ERI-6/7 Helicase Acts at the First Stage of an siRNA Amplification Pathway That Targets Recent Gene Duplications
Source: PLoS Genet. 2011 Nov 10;7(11):e1002369. doi: 10.1371/journal.pgen.1002369 (PMC3213143; doi:10.1371/journal.pgen.1002369)
Supplement: Table S1 — Deep sequencing statistics. (DOC) [file pgen.1002369.s008.doc]

**Table S1**. Deep sequencing statistics

| Sequence data | N2  embryo | *eri-1(mg366)* embryo | *eri-7(mg369)* embryo | *eri-6(mg379)* embryo | *ergo-1(tm1860)* embryo | N2  adult | *eri-7(mg369)*  adult |
| --- | --- | --- | --- | --- | --- | --- | --- |
| Processed | 22884703 | 22283068 | 21593067 | 20035226 | 21509805 | 9029082 | 8203677 |
| Parsed | 21383541 | 21821888 | 20588630 | 19794958 | 21231406 | 7939402 | 7106690 |
| Mapped | 18309931 | 18507557 | 17576340 | 17931342 | 18714322 | 6413058 | 5757891 |
| Unique | 1141561 | 1302788 | 1141684 | 486040 | 671898 | 2089895 | 1778940 |
