# Supplementary material for: The ERI-6/7 Helicase Acts at the First Stage of an siRNA Amplification Pathway That Targets Recent Gene Duplications
Source: PLoS Genet. 2011 Nov 10;7(11):e1002369. doi: 10.1371/journal.pgen.1002369 (PMC3213143; doi:10.1371/journal.pgen.1002369)
Supplement: Table S4 — Gene products eri-6/7-dependent siRNA target genes are relatively poorly conserved in the related nematode C. briggsae and in H. sapiens. (DOC) [file pgen.1002369.s011.doc]

**Table S4.** Conservation of *eri-6/7* target gene products in *C. briggsae* and *H. sapiens*.

| organism | #endo-siRNA target genes with homolog | median E-value of best homologs (endo-siRNA targets) | median E-value of 26,533 best homologs (whole genome) | average ranking (from low E value to high E value) of target gene products | total number of best homologs in *C. elegans* genome with E value<0.1 |
| --- | --- | --- | --- | --- | --- |
| *C. briggsae* | 34 | 5.9E-07 | 5.8E-114 | 25,102 | 26,533 |
| *H. sapiens* | 27 | 7.4E-03 | 1.3E-25 | 18,291 | 21,745 |
