# Supplementary material for: The ERI-6/7 Helicase Acts at the First Stage of an siRNA Amplification Pathway That Targets Recent Gene Duplications
Source: PLoS Genet. 2011 Nov 10;7(11):e1002369. doi: 10.1371/journal.pgen.1002369 (PMC3213143; doi:10.1371/journal.pgen.1002369)
Supplement: Table S5 — eri-6/7-dependent siRNA target genes consist of relatively few exons and are short. (DOC) [file pgen.1002369.s012.doc]

**Table S5. *eri-6/7* target gene length and structure.**

|  | median number of exons | average number of exons | median gene length | average gene length |
| --- | --- | --- | --- | --- |
| endo-siRNA target genes (47) | 3 | 3.7 | 1,051 | 1,295 |
| all *C. elegans* genes (20,163) | 5 | 6.2 | 1,826 | 2,818 |
| P-value t-test | 3.8*10^-5^ |  | 2.8*10^-3^ |  |
