# Supplementary material for: The ERI-6/7 Helicase Acts at the First Stage of an siRNA Amplification Pathway That Targets Recent Gene Duplications
Source: PLoS Genet. 2011 Nov 10;7(11):e1002369. doi: 10.1371/journal.pgen.1002369 (PMC3213143; doi:10.1371/journal.pgen.1002369)
Supplement: Table S6 — Proteomics analysis of mixed stage C. elegans [35] shows that few eri-6/7 target genes produce proteins. (DOC) [file pgen.1002369.s013.doc]

**Table S6.** Proteomics analysis of mixed stage *C. elegans* (Schrimpf *et al.* (2009)) shows that few *eri-6/7* target genes produce proteins.

|  | # peptides identified | #unique peptides | #genes producing protein |
| --- | --- | --- | --- |
| whole genome | 759,320 | 84,962 | 10,631 out of 19,735 (54%) |
| *eri-6/7* targets | 34 | 26 | 16 out of 93 (17%) |
