# Supplementary material for: The ERI-6/7 Helicase Acts at the First Stage of an siRNA Amplification Pathway That Targets Recent Gene Duplications
Source: PLoS Genet. 2011 Nov 10;7(11):e1002369. doi: 10.1371/journal.pgen.1002369 (PMC3213143; doi:10.1371/journal.pgen.1002369)
Supplement: Table S7 — Brood size of eri-6/7 mutants. (DOCX) [file pgen.1002369.s014.docx]

**Table S7.** Brood size of *eri-6/7* mutants.

|  | 15 °C | 20 °C | 25 °C |
| --- | --- | --- | --- |
| wild type | 224 ± 41 | 292 ± 74 | 198 ± 50 |
| *eri-6(mg379)* | 169 ± 30 | 192 ± 26 | 79 ± 17 |
| *eri-7(mg369)* | 206 ± 37 | 148 ± 61 | 72 ± 20 |
