# Supplementary material for: The ERI-6/7 Helicase Acts at the First Stage of an siRNA Amplification Pathway That Targets Recent Gene Duplications
Source: PLoS Genet. 2011 Nov 10;7(11):e1002369. doi: 10.1371/journal.pgen.1002369 (PMC3213143; doi:10.1371/journal.pgen.1002369)
Supplement: Table S8 — Enhanced RNAi and transgene silencing phenotypes of eri-6 and eri-6;nrde-3 double mutants. (DOC) [file pgen.1002369.s015.doc]

**Table S8**. Eri and transgene silencing phenotypes of *eri-6* and *eri-6;nrde-3* double mutants upon RNAi to the genes *hmr-1, dpy-13* and *unc-73* or of transgenic animals carrying a transgene with a dominant *rol-6* mutation. Scored phenotypes: Emb (embryonic lethal), Dpy (dumpy), Unc (uncoordinated), Rol (rolling movement).

| genotype | *hmr-1(RNAi)* | *dpy-13(RNAi)* | *unc-73(RNAi)* | *rol-6* transgene |
| --- | --- | --- | --- | --- |
| *wild type* | not Emb | medium Dpy | not Unc | 100% Rol |
| *eri-6(mg379)* | 100% Emb | strong Dpy | strong Unc | 100% non-Rol |
| *nrde-3(tm1116)* | n/a | n/a^1^ | n/a^1^ | 50% non-Rol |
| *eri-6(mg379); nrde-3(tm1116)* | 100% Emb | medium Dpy | strong Unc | 100% Rol |

*^1^: nrde-3(gg066)* was previously shown to respond similar as wild type to *dpy-13*(RNAi) and *unc-73*(RNAi)
