# Supplementary material for: The ERI-6/7 Helicase Acts at the First Stage of an siRNA Amplification Pathway That Targets Recent Gene Duplications
Source: PLoS Genet. 2011 Nov 10;7(11):e1002369. doi: 10.1371/journal.pgen.1002369 (PMC3213143; doi:10.1371/journal.pgen.1002369)
Supplement: Table S10 — Primers used in this study. (DOC) [file pgen.1002369.s017.doc]

**Table S10.** Primers used in this study.

| gene |  | sequence |
| --- | --- | --- |
| ZK380.5 | forward | agattgtctgcgacatgcac |
| ZK380.5 | reverse | gacatggtggcacttgaatg |
| T08B6.2 | forward | cccgaacaaacggaatatgt |
| T08B6.2 | reverse | gagtgacccgagaccagaaa |
| F55C9.3 | forward | ggaaaaccggaatcattcaa |
| F55C9.3 | reverse | agctccacgttgtagcgtct |
| Y45F10D.4 | forward | gtcgcttcaaatcagttcagc |
| Y45F10D.4 | reverse | gttcttgtcaagtgatccgaca |
| *rpl-32* | forward | caaggtcgtcaagaagaagc |
| *rpl-32* | reverse | ggctacacgacggtatctgt |
